# Long-term outcome of cardiac resynchronization therapy patients in the elderly

**DOI:** 10.1007/s11357-023-00739-z

**Published:** 2023-02-17

**Authors:** Anett Behon, Eperke Dóra Merkel, Walter Richard Schwertner, Luca Katalin Kuthi, Boglárka Veres, Richard Masszi, Attila Kovács, Bálint Károly Lakatos, Endre Zima, László Gellér, Annamária Kosztin, Béla Merkely

**Affiliations:** Heart and Vascular Center, Semmelweis University, Varosmajor 68 H-1122, Budapest, Hungary

**Keywords:** Cardiac resynchronization therapy, Elderly, Age-related differences

## Abstract

Heart failure (HF) is a leading cause of mortality and hospitalization in the elderly. However, data are scarce about their response to device treatment such as cardiac resynchronization therapy (CRT). We aimed to evaluate the age-related differences in the effectiveness of CRT, procedure-related complications, and long-term outcome. Between 2000 and 2020, 2656 patients undergoing CRT implantation were registered and analyzed retrospectively. Patients were divided into 3 groups according to their age: group I, < 65; group II, 65–75; and group III, > 75 years. The primary endpoint was the echocardiographic response defined as a relative increase > 15% in left ventricular ejection fraction (LVEF) within 6 months, and the secondary endpoint was the composite of all-cause mortality, heart transplantation, or left ventricular assist device implantation. Procedure-related complications were also assessed. After implantation, LVEF showed significant improvement both in the total cohort [28% (*IQR* 24/33) vs. 35% (*IQR* 28/40); *p* < 0.01)] and in each subgroup (27% vs. 34%; *p* < 0.01, 29% vs. 35%; *p* < 0.01, 30% vs. 35%; *p* < 0.01). Response rate was similar in the 3 groups (64% vs. 62% vs. 56%; *p* = 0.41). During the follow-up, 1574 (59%) patients died. Kaplan–Meier curves revealed a significantly lower survival rate in the older groups (log-rank *p* < 0.001). The cumulative complication rates were similar among the three age groups (27% vs. 28% vs. 24%; *p* = 0.15). Our results demonstrate that CRT is as effective and safe therapy in the elderly as for young ones. The present data suggest that patients with appropriate indications benefit from CRT in the long term, regardless of age.

**Supplementary Information:**

The online version contains supplementary material available at 10.1007/s11357-023-00739-z.

## Introduction

Heart failure (HF) is one of the leading causes of mortality and hospitalization in the elderly [1, 2]. The prevalence of HF increases sharply with age; it affects approximately 1–2% of the adult population in developed countries, reaching up to 10% among people above 70 years of age [3]. Elderly HF patients are considered a vulnerable population with a high chance of polypharmacy, multimorbidity, cognitive decline, and frailty [4]. Despite these data, there are no specific guidelines for HF management in the elderly population [5, 6].

Besides pharmacotherapy, several randomized controlled trials (RCTs) have shown that cardiac resynchronization therapy (CRT) reduces morbidity and mortality in symptomatic HF patients with reduced left ventricular ejection fraction (HFrEF) and a wide QRS [7–15]. However, in most RCTs, the number of enrolled elder patients is limited resulting in an age discrepancy, and those who are enrolled in studies rather have fewer comorbidities compared with the real-world CRT population [16]. These differences may influence the outcome as well. Since these trials have such limitations, real-world data may address the question of the rate of CRT response in the elderly [6, 7, 9, 17].

More observational trials’ data confirmed that the mortality rate among patients ≥ 75 years was higher than in younger patients mainly due to non-cardiac causes [18, 19], but the incidence of HF hospitalization was similar by age [20]. Moreover, the modes of death in HF patients have been changing due to aging and effective pharmacological treatments, as HF patients die less commonly in sudden cardiac death or HF progression; the decreasing mortality rate due to cardiovascular causes can result in a shift to non-cardiovascular death, which reaches almost 50% in the elderly HF patients [21, 22]. The association between older age and adverse events highlights the importance of assessing the efficacy and safety of CRT before implantation and the need for real-world data.

Based on these effects, it is an important question, whether age may negatively affect the CRT response in this elder and ever-growing population of CRT candidates [3, 23, 24].

Therefore, the aim of our study was to evaluate the age-related differences in the effectiveness of CRT, peri- and postprocedural complications, and long-term outcome after CRT implantation in a large-scale, real-world single-center registry.

## Methods

### Patients and follow-up

From October 2000 to September 2020, patients undergoing successful CRT implantation, as per current guidelines, at the Heart and Vascular Center, Semmelweis University, Budapest, Hungary, were retrospectively registered in our *Biobankok* database [3, 25–27]. The registry enrolled patients with symptomatic chronic systolic HF (NYHA II–IVa), reduced LVEF (*EF* ≤ 35%), and a prolonged QRS (*QRS* ≥ 130 ms).

For each patient baseline clinical characteristics such as demographic data, medical history, physical status, medical therapy, ECG, echocardiographic, and laboratory parameters were collected retrospectively from paper-based or electronic medical records at the time of implantation and up to 6 months after the procedure.

To evaluate the association between age and the effect of CRT, peri- and postprocedural complications, and long-term outcome, patients were divided into 3 groups according to their age at the time of the implantation: group I, < 65; group II, 65–75; and group III, > 75 years as defined in previous studies [28, 29].

The status of our patients was updated in December 2021 from the National Health Insurance of Hungary Database, which provided us with the exact date of death. The study protocol complies with the Declaration of Helsinki, and the protocol was approved by the Medical Research Council; ETT- TUKEB No. 161–0/2019.

### CRT implantation procedure

Device implantations were performed according to the current standards by using a transvenous approach or transseptal method. During device implantation, all left ventricular lead placements were tailored by coronary sinus venogram during the fluoroscopy. After successful positioning of the leads, electrical parameters such as pacing, sensing, and impedance values were measured and also registered in the Biobankok system.

### Study endpoints

The primary endpoint was echocardiographic response to CRT. The improvement in left ventricular ejection fraction (LVEF) was assessed as a continuous variable. Reverse remodeling was defined as a relative increase of 15% or more in LVEF within 6 months after CRT implantation.

Secondary endpoint was the composite of all-cause mortality or heart transplantation (HTX) or implantation of a left ventricular assist device (LVAD) during long-term follow-up investigated by age groups.

Tertiary endpoints were peri- and postprocedural complications in the three groups. Time-trend effects on age, device types, and response rate were also assessed.

### Statistical analysis

Continuous variables with a parametric distribution are summarized as the mean and standard deviation (SD), while those with non-parametric distributions are presented as medians with interquartile range (IQR). Categorical variables are expressed as numbers and percentages (*n*, %). Baseline clinical characteristics were compared by unpaired *t*-test for normally distributed continuous variables; for not normally distributed continuous variables, the Mann–Whitney test was used. Comparisons between three groups of patients were performed using one-way ANOVA test or Kruskal–Wallis test for normally and not normally distributed continuous variables, respectively. For categorical variables, *χ*2-test or Fisher exact test was performed, as appropriate.

Survival after device implantation was presented using Kaplan–Meier curves using the log-rank test. Unadjusted hazard ratios (HR) with 95% confidence intervals (95% CI) were calculated in Cox proportional hazards models to evaluate the impact of age (group I vs. group II; group I vs. group III; group II vs. group III) on the secondary composite endpoint. A two-sided *p*-value of < 0.05 was considered statistically significant.

We modeled age as a continuous variable to better characterize the shape of the association of higher age with all-cause mortality using proportional hazards regression restricted cubic spline models with knots located at each age value. All statistical analyses were performed using GraphPad Prism version 8.0 (GraphPad Inc., CA, USA) and the SPSS v21 software (IBM, NY, USA).

## Results

### Baseline clinical characteristics

Altogether 2656 patients underwent successful CRT implantation and were enrolled in the current analysis. In the total cohort, 1028 (39%) patients were < 65 years old [median 59, (*IQR* 53/62) years], 1004 (38%) were between the ages of 65 and 75 [median 70 (*IQR* 68/72) years], and 624 (23%) patients were > 75 years old [median 79 (*IQR* 77/82) years] (Table 1). Most of the patients in each age group were male (group I: 78% vs. group II: 74% vs. group III: 72%), approximately 75% of the total cohort. However, the percentage of women significantly increased with age (group I: 22% vs. group II: 26% vs. group III: 28%; *p* < 0.01).

**Table 1:** Baseline clinical characteristics of patients by age

Baseline variables	All patients (*n* = 2656)	< 65 years old (*n* = 1028)	65–75 years old (*n* = 1004)	> 75 years old (*n* = 624)	*p* value
Age (yrs; median/IQR)	68 (61–75)	59 (53–62)	70 (68–72)	79 (77–82)	< 0.01****
Gender (female; *n*; %)	667 (25%)	224 (22%)	266 (26%)	177 (28%)	< 0.01**
NYHA III/IV (st; *n*; %)	1237 (46%)	490 (48%)	469 (47%)	277 (44%)	0.43
Ischemic etiology (*n*; %)	1296 (49%)	415 (40%)	520 (52%)	361 (58%)	< 0.01****
CRT-D (*n*; %)	1452 (55%)	614 (60%)	563 (56%)	275 (44%)	< 0.01****
RR systolic (mmHg; median/*IQR*)	125 (111–138)	120 (111–133)	125 (111–136)	130 (115–143)	< 0.01****
RR diastolic (mmHg; median/*IQR*)	73 (65–80)	74 (68–81)	72 (64–80)	72 (65–80)	0.03*
BMI (kg/m^2^; median/*IQR*)	27.4 (24.6–30.8)	27.8 (24.8–31.4)	27.7 (24.7–31)	26.8 (24.2–29.4)	< 0.01****
QRS (ms; median/*IQR*)	160 (140–180)	160 (140–175)	160 (146–180)	160 (144–178)	0.19
LBBB morphology (*n*; %)	1838 (69%)	734 (71%)	673 (67%)	431 (69%)	0.10
Medical history					
Atrial fibrillation (*n*; %)	1014 (38%)	318 (31%)	415 (41%)	281 (45%)	< 0.01***
Diabetes mellitus (*n*; %)	964 (36%)	346 (34%)	399 (40%)	219 (35%)	0.01*
Type 2 DM (*n*; %)	782 (29%)	282 (27%)	326 (32%)	174 (28%)	0.03*
Hypertension (*n*; %)	1916 (72%)	655 (64%)	764 (76%)	497 (79%)	< 0.01***
Prior MI (*n*; %)	1009 (38%)	337 (33%)	400 (40%)	272 (43%)	< 0.01***
Prior PCI (*n*; %)	786 (29%)	235 (23%)	319 (32%)	232 (37%)	< 0.01***
Prior CABG (*n*; %)	354 (13%)	109 (11%)	142 (14%)	103 (16%)	< 0.01**
Prior COPD (*n*; %)	393 (15%)	144 (14%)	162 (16%)	87 (14%)	0.32
Laboratory parameters					
Serum urea (µmol/l; median/*IQR*)	403 (321–489)	399 (330–495)	407 (320–480)	397 (304–478)	0.32
Serum creatinine (µmol/l; median/*IQR*)	101 (82–130)	93 (78–120)	103 (84–133)	110 (87–140)	< 0.01***
Serum cholesterol (mmol/l; median/*IQR*)	4.1 (3.4–5.1)	4.3 (3.4–5.4)	4.2 (3.5–5.0)	3.9 (3.1–4.7)	< 0.01***
eGFR (ml/min/1.73m^2^; median/*IQR*)	63.7 (47.7–80.7)	71.2 (54.6–88.2)	61.3 (45.7–76.5)	56.8 (41.9–72.2)	< 0.01****
NT-proBNP (pmol/l; median/*IQR*)	2748 (1454–4146)	2587 (1367–3810)	2517 (1444–3550)	3000 (1690–4714)	0.03*
Echocardiographic parameters					
LVEF (%; median/*IQR*)	28 (24–33)	27 (22–32)	29 (25–33)	30 (25–35)	< 0.01****
LVEDV (ml; median/*IQR*)	216 (166–280)	236 (187–305)	210 (157–262)	187 (142–245)	< 0.01****
LVESV (ml; median/*IQR*)	160 (118–210)	177 (131–227)	153 (119–201)	129 (103–178)	< 0.01***
LVEDD (mm; median/*IQR*)	63 (57–70)	66 (60–73)	63 (57–69)	61 (55–66)	< 0.01****
LVESD (mm; median/*IQR*)	53 (47–60)	56 (49–63)	53 (46–60)	50 (43–56)	< 0.01****
Medical treatment					
Beta blocker (*n*;%)	2161 (81%)	831 (81%)	819 (81%)	511 (82%)	0.83
ACE-I/ARB (*n*;%)	2233 (84%)	847 (82%)	858 (85%)	528 (85%)	0.15
MRA (*n*;%)	1652 (62%)	662 (64%)	621 (62%)	369 (59%)	0.10
Furosemide (*n*;%)	1927 (72%)	709 (69%)	742 (74%)	476 (76%)	< 0.01**
Digoxin (*n*; %)	491 (18%)	220 (21%)	186 (18%)	85 (14%)	< 0.01***
Amiodarone (*n*;%)	656 (25%)	250 (24%)	274 (27%)	132 (21%)	0.02*
Oral anticoagulant therapy (*n*;%)	838 (31%)	293 (28%)	329 (33%)	215 (34%)	0.03*

*ACE-I*, angiotensin converting enzyme inhibitors; *ARB*, angiotensin receptor blocker, *BMI*, body mass index; *CABG*, coronary artery bypass grafting; *COPD*, chronic obstructive pulmonary disease; *CRT-D*, cardiac resynchronization therapy defibrillator; *DM*, diabetes mellitus; *eGFR*, estimated glomerular filtration rate; *LBBB*, left bundle branch block; *LVEDD*, left ventricular end-diastolic diameter; *LVEDV*, left ventricular end-diastolic volume; *LVEF*, left ventricular ejection fraction; *LVESD*, left ventricular end-systolic diameter; *LVESV*, left ventricular end-systolic volume; *MI*, myocardial infarction; *MRA*, mineralocorticoid receptor antagonists; *NT-proBNP*, N-terminal pro-B-type natriuretic peptide; *NYHA*, New York Heart Association class; *PCI*, percutaneous coronary intervention

^*^ *p* < 0.05, ** *p* < 0.01, *** *p* < 0.001, **** *p* < 0.0001

Older patients were more likely to have ischemic etiology (group III: 58% vs. group II: 52% vs. group I: 40%; *p* < 0.01), higher systolic blood pressure [group III: median 130 (*IQR* 115/143) Hgmm vs. group II: median 125 (*IQR* 111/136) Hgmm vs. group I: median 120 (IQR 111/133) Hgmm; *p* < 0.01], and lower BMI [group III: median 26.8 (*IQR* 24.2/29.4) kg/m^2^ vs. group II: median 27.7 (*IQR* 24.7/31) kg/m^2^ vs. group I: median 27.8 (*IQR* 24.8/31.4) kg/m^2^; *p* < 0.01] and were more frequently implanted with a cardiac resynchronization therapy pacemaker (CRT-P) (group III: 56% vs. group II: 44% vs. group I: 40%; *p* < 0.01) (Table 1).

In laboratory parameters, older participants had higher serum creatinine levels [group III: median 110 (*IQR* 87/140) µmol/l vs. group II: median 103 (*IQR* 84/133) µmol/l vs. group I: median 93 (*IQR* 78/120) µmol/l; *p* < 0.01], lower eGFR (group III: median 56.8 (*IQR* 41.9/72.2) ml/min/1.73m^2^ vs. group II: median 61.3 (*IQR* 45.7/76.5) ml/min/1.73m^2^ vs. group I: median 71.2 (*IQR* 54.6/88.2) ml/min/1.73m^2^; *p* < 0.01], lower cholesterol levels (group III: median 3.9 mmol/l vs. group II: median 4.2 mmol/l vs. group I: median 4.3 mmol/l; *p* < 0.01), and similar serum urea levels [group III: median 397 (*IQR* 304/478) µmol/l vs. group II: median 407 (*IQR* 320/480) µmol/l vs. group I: median 399 (*IQR* 330/495) µmol/l; *p* = 0.32], (Table 1).

Age-related differences could be also detected in echocardiographic parameters. Older patients had higher LVEF [group III: median 30 (*IQR* 25/35) % vs. group II: median 29 (*IQR* 25/33) % vs. group I: median 27 (*IQR* 22/32) %; *p* < 0.01], lower left ventricular end-diastolic (LVEDV) [group III: median 187 (*IQR* 142/245) ml vs. group II: median 210 (*IQR* 157/262) ml vs. group I: median 236 (*IQR* 187/305) ml; *p* < 0.01] and end-systolic volume (LVESV) [group III: median 129 (*IQR* 103/178) ml vs. group II: median 153 (*IQR* 119/201) ml vs. group I: median 177 (*IQR* 131/227) ml; *p* < 0.01], and lower left ventricular end-diastolic (LVEDD) [group III: median 61 (*IQR* 55/66) mm vs. group II: median 63 (*IQR* 57/69) mm vs. group I: median 66 (*IQR* 60/73) mm; *p* < 0.01] and end-systolic diameter (LVESD) [group III: median 50 (*IQR* 43/56) mm vs. group II: median 53 (*IQR* 46/60) mm vs. group I: median 56 (*IQR* 49/63) mm; *p* < 0.01] (Table 1).

Regarding comorbidities, older patients more frequently had atrial fibrillation (group III: 45% vs. group II: 41% vs. group I: 31%; *p* < 0.01), diabetes mellitus (group III: 35% vs. group II: 40% vs. group I: 34%; *p* = 0.01), hypertension (group III: 79% vs. group II: 76% vs. group I: 64%; *p* < 0.01), prior myocardial infarction (group III: 43% vs. group II: 40% vs. group I: 33%; p < 0.01), percutaneous coronary intervention (PCI) (group III: 37% vs. group II: 32% vs. group I: 23%; *p* < 0.01), and coronary artery bypass grafting (CABG) (group III: 16% vs. group II: 14% vs. group I: 11%; *p* < 0.01) (Table 1). As regards medical therapy, each subgroup was added optimal heart failure basic treatment at a similarly high rate; at the same time, older patients were more likely to be treated with loop diuretics (group III: 76% vs. group II: 74% vs. group I: 69; *p* < 0.01), amiodarone (group III: 21% vs. group II: 27% vs. group I: 24%; *p* = 0.02), and oral anticoagulant therapy (group III: 34% vs. group II: 33% vs. group I: 28%; p = 0.03), less likely with digoxin (group III: 14% vs. group II: 18% vs. group I: 21%; *p* < 0.001) (Table 1).

### Primary endpoint: echocardiographic response by age groups

After CRT implantation, LVEF showed a significant improvement in the whole population [median 28 (*IQR* 24/33) % at baseline vs. median 35 (*IQR* 28/40) % at 6 months; *p* < 0.01] as well as in each subgroup [group I: median 27 (*IQR* 22/32) % vs. median 34 (*IQR* 28/40) %; *p* < 0.01; group II: median 29 (*IQR* 25/33) % vs. median 35 (*IQR* 29/40) %; *p* < 0.01; group III: median 30 (*IQR* 25/35) % vs. median 35 (*IQR* 29/43) %; *p* < 0.01] (Table 2). The percentage of responders was comparable between the three groups, 64% in group I, 61% in group II, and 56% in group III (*p* = 0.41) (Table 3).

**Table 2:** Echocardiographic response divided by age groups

	< 65 years old	65–75 years old	> 75 years old
Baseline LVEF (%; median/*IQR*)	27 (22/32)	29 (25/33)	30 (25/35)
6 months LVEF (%; median/*IQR*)	34 (28/40)	35 (29/40)	35 (29/43)
*p*-value	< 0.01****	< 0.01****	< 0.01****

*LVEF*, left ventricular ejection fraction

^****^ *p* < 0.0001

**Table 3:** Reverse remodeling divided by age groups

	< 65 years old *n* = 201	65–75 years old *n* = 195	> 75 years old *n* = 114	*p*-value
Reverse remodeling*	128 (64%)	120 (61%)	64 (56%)	0.41

^*^Reverse remodeling was defined as a relative increase of 15% or more in left ventricular ejection fraction within 6 months after CRT implantation

### Secondary endpoint: long-term all-cause mortality by age groups

**Fig. 1:**
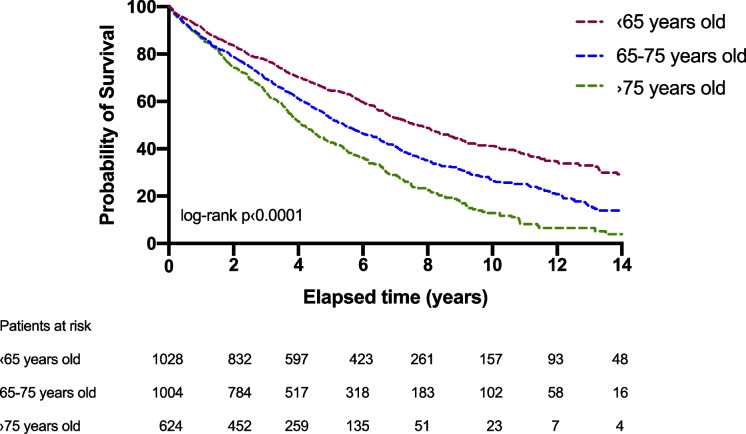
Kaplan–Meier estimates of the probability of survival by age groups

**Fig. 2:**
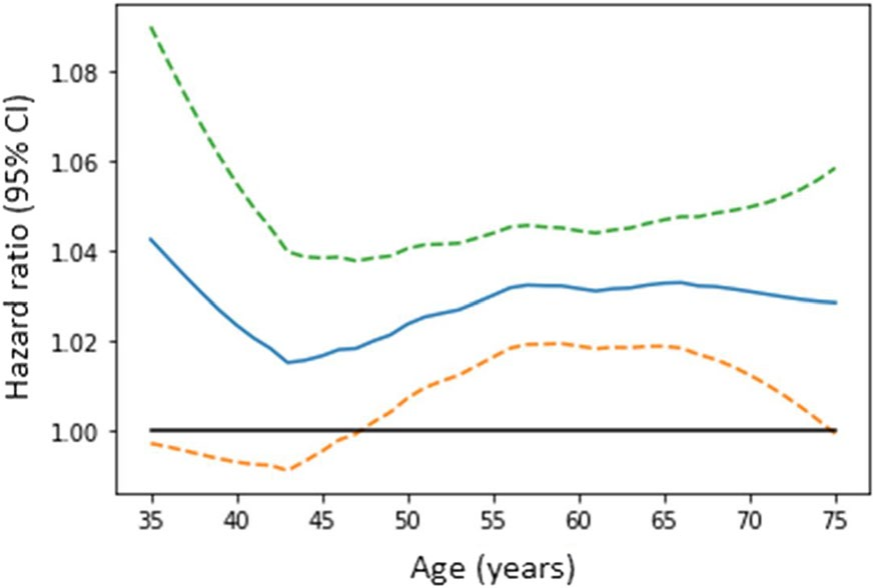
Restricted cubic spline regression for the association between age and risk of all-cause mortality

During long-term follow-up (median 4.1 years interquartile range 2.3–6.9 years), a total of 1574 (57%) patients reached the secondary composite endpoint, 511 (50%) in group I, 637 (63%) in group II, and 426 (68%) in group III. Kaplan–Meier curves revealed a significantly lower survival rate in the older groups compared to the younger ones (log-rank *p* < 0.001) (Fig. 1) (Table 4). Restricted cubic spline based on Cox regression was used to flexibly model the association between age and all-cause mortality risk shown in Fig. 2. The lowest inflection point was found around 43 years.

**Table 4:** The associations of age with the risk of all-cause mortality

Comparison of different age groups			
End point	All-cause mortality		
	Hazard ratio	*95% CI*	*p*-value
< 65 yrs vs. 65–75 yrs	0.67	0.60–0.76	< 0.01****
< 65 yrs vs. > 75 yrs	0.51	0.44–0.58	< 0.01****
65–75 yrs vs. > 75 yrs	0.72	0.64–0.82	< 0.01****

^****^ *p* < 0.0001

### Tertiary endpoints: peri- and postprocedural complications by age groups

Peri- and postprocedural complications were observed in 710 (27%) patients (Table 5). The most frequent events were lead dislodgement (7%) and phrenic nerve stimulation (5%). No statistical differences were observed in any complications among the three age groups. Numerically pocket infection/decubitus (group III: 1% vs. group II: 2% vs. group I: 3%; *p* = 0.04) and sepsis (group III: 0.2% vs. group II: 0.1% vs. group I: 5.3%; *p* < 0.001) were observed less frequently in older patients but in a very low incidence in the total cohort (Table 5).

**Table 5:** Peri-procedural complications divided by age groups

Complications	All patients (*n* = 2656)	< 65 years old (*n* = 1028)	65–75 years old (*n* = 1004)	> 75 years old (*n* = 624)	*p*-value
All complications (*n*;%)	710 (27%)	275 (27%)	285 (28%)	150 (24%)	0.15
Bleeding (*n*;%)	39 (1.5%)	13 (1.2%)	19 (1.9%)	7 (1%)	0.36
Pneumothorax (*n*;%)	32 (1.2%)	11 (1.1%)	11 (1.1%)	10 (1.6%)	0.58
Hemothorax (*n*;%)	5 (0.2%)	0 (0%)	3 (0.3%)	2 (0.3%)	0.20
Coronary sinus dissection (*n*;%)	25 (0.9%)	14 (1.4%)	5 (0.5%)	6 (0.9)	0.13
Pericardial tamponade (*n*;%)	10 (0.4%)	4 (0.4%)	1 (0.1%)	5 (0.8%)	0.08
Pocket infection/decubitus (*n*;%)	67 (2.5%)	36 (3.5%)	21 (2.1%)	10 (1.6%)	0.03*
Infective endocarditis (*n*;%)	16 (0.6)	6 (0.6%)	8 (0.8)	2 (0.3%)	0.48
Lead dislodgement (*n*;%)	178 (7%)	69 (6.7%)	75 (7.5%)	34 (5.4%)	0.28
Lead dysfunction/fracture (*n*;%)	48 (2%)	20 (2%)	21 (2%)	7 (1%)	0.33
Sepsis (*n*;%)	7 (0.2)	55 (5.3%)	1 (0.1)	1 (0.2)	< 0.01****
Phrenic nerve stimulation (*n*;%)	142 (5%)	45 (4%)	66 (7%)	31 (5%)	0.08

^*^ *p* < 0.05, **** *p* < 0.0001

### Time-trend effects on age, the use of device types, and response rate in the elderly

**Fig. 3:**
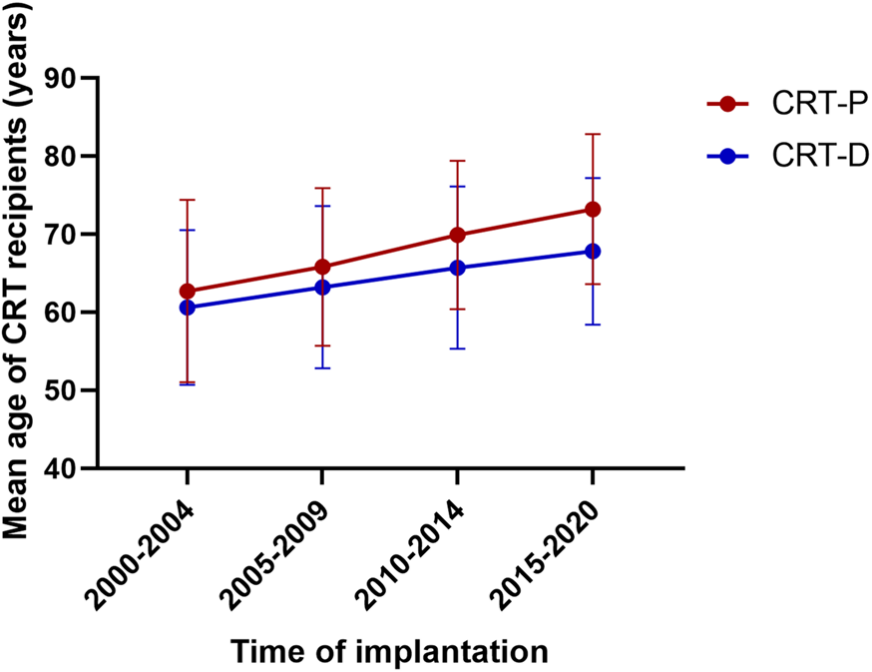
The mean age of CRT recipients within 5-year intervals

**Fig. 4:**
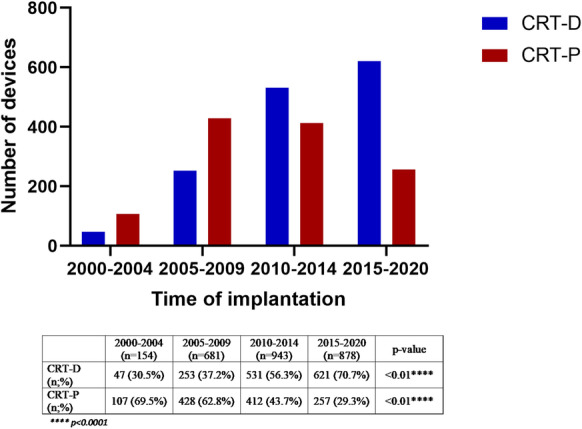
Number of CRT-D and CRT-P implantations within 5-year intervals

**Fig. 5:**
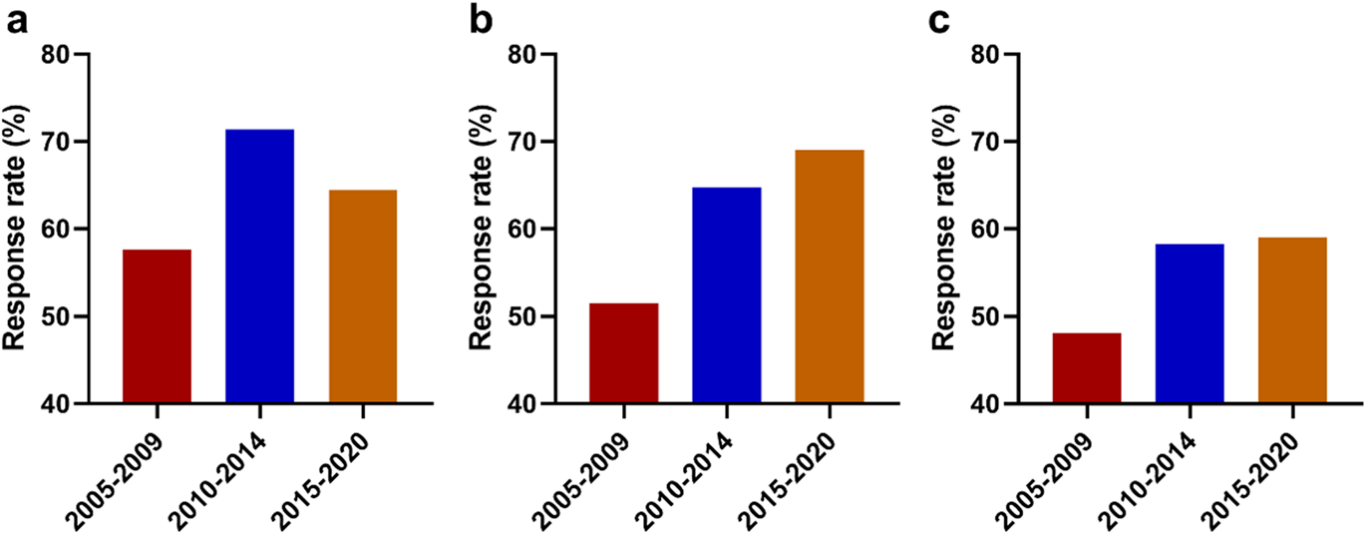
Response rate within 5-year intervals in group I (**a**), group II (**b**), and group III (**c**)

The mean age of CRT recipients increased significantly over the last 20 years analyzed by 5-year intervals: 2000–2004, 62.0 ± 11.2 years; 2005–2009, 64.8 ± 10.2 years; 2010–2014, 67.6 ± 10.2 years; 2015–2020, 69.3 ± 9.8 years; *p* < 0.01 (Fig. 3). Assessing the type of the device, we observed that the mean age of CRT-P and cardiac resynchronization therapy with defibrillator (CRT-D) patients both increased significantly since the early 2000s and that the mean age of CRT-P patients was significantly higher than that of CRT-D patients in each subgroup, except for 2000–2004 (Supplementary Table 1 and 2.). With regard to the device type, there was a significant increase in the percentage of CRT-D implantation: 2000–2004, 30.5%; 2005–2009, 37.2%; 2010–2014, 56.3%; 2015–2020, 70.7%; *p* < 0.01 (Fig. 4). The response rate increased over time in each subgroup but did not reach statistical significance (Fig. 5).

## Discussion

Our present analysis demonstrates that older patients benefit from CRT implantation similar to younger patients according to their clinical outcome, with comparable improvement in LV function and similar rates of peri-, and postprocedural complications. However, older patients showed significantly higher all-cause mortality risk compared to younger ones; this difference can be explained by the higher rate of comorbidities among older participants.

Time-trend investigations showed an increasing mean age of both CRT-P and CRT-D patients since 2000 and a significant difference between the mean age of these two subgroups. Regarding the type of devices, we found a significant increase in the rate of CRT-D implantation. Moreover, the response rate also increased over time in each age group, but did not reach statistical significance.

### Baseline clinical characteristics

Baseline clinical characteristics of our patient population were consistent with variables of patients enrolled in previous RCTs and real-world evidence studies, analyzing age-related differences in CRT response and outcome [8, 9, 18–20, 28–44]. In the older age groups, a predominance of the female sex, ischemic etiology, and worse renal function, but higher LVEF and smaller LV chamber sizes, could be observed. Regarding comorbidities, older patients were more likely to have multiple concomitant chronic illnesses, which may affect the prognosis of HF and cause a higher incidence of non-cardiovascular death in the elderly [6, 21]. In a previous study, Braunstein et al. evaluated the impact of non-cardiac comorbidities on clinical outcomes in HF patients and found that the number of these concomitant diseases was directly related to the number of hospitalization and mortality rate [45]. Several other studies analyzed the impact of specific comorbidities on clinical outcomes in HF patients, reporting that the presence of renal insufficiency, anemia, and impaired cognition are associated with adverse outcomes [46–49]. After CRT implantation, chronic renal failure, diabetes mellitus, and atrial fibrillation were found to be strong independent predictors of death [50, 51].

### Response to CRT

Since the number of older HF patients is increasing due to the aging population and significant prolongation of life, it is an important issue, whether age negatively affects the response to CRT [3, 23, 24]. CRT induces LV reverse remodeling and improvement in LV systolic function, which have been shown to be independent of age both in RCTs and real-world evidence studies [18–20, 28, 29, 32, 34, 35, 38–41]. Several previous studies showed comparable improvement in LVEF and LV dimensions after CRT implantation regardless of age. The MIRACLE and MIRACLE-ICD trials found no differences by age in LVEF improvement [28]. Similarly, the substudy of the InSync/InSync ICD Italian Registry showed that CRT induced significant and similar left ventricular reverse remodeling, resulting in a comparable responder rate in the 3 age groups [19]. In our retrospective analysis, 6 months after CRT implantation, LVEF showed a significant improvement in the whole population as well as in each subgroup. The percentage of response to CRT was not significantly different between the three age groups. Our results are in line with the findings of previous studies, demonstrating that the response to CRT is not affected by advanced age [18–20, 28, 29, 32, 34, 35, 38–41]. Based on the findings of our recent and these previous studies, the full age range of patients with advanced heart failure seems to benefit from CRT. However, in different cohorts, age can be numerically the same, and the expected response might be associated with rather the frailty, which also involves the biological status of the individuals.

### Device-related complications

Regarding procedure-related complications, several observational trials and subgroup analyses found no statistical difference between the different age groups [18, 19, 28–30, 34, 35, 39, 40, 43]. These findings are in line with our results demonstrating that CRT implantation is a safe and well-tolerated procedure even in the elderly. Nevertheless, most of the RCTs and large-scale observational trials involve high-volume centers, which may lead to a lower rate of complications. As pneumothorax is also described as a more frequent adverse event in the elderly during CRT implantation compared to younger patients[21], we could not confirm it. In our present analysis, we observed inequality in the rate of pocket infections among the elderly and younger ones; the lower rate in older patients may associate with the lower prevalence of this complication in both groups.

### Long-term outcome

The long-term outcome of patients with HF depends on many variables, such as electrical parameters during the implantation, sex, age, and frailty [52–55]. The subgroup analyses of RCTs and previous observational studies found that the risk of all-cause mortality or hospitalization for HF and HF hospitalization alone is independent of age [8, 9, 18–20, 32, 35, 37, 38, 41, 43, 44]. Still, investigating all-cause mortality alone, most of the previous studies reported a significantly higher mortality rate among older patients [18–20, 29, 30, 38, 42]. With modern therapy options and age, the mode of death in HF patients has changed; less frequently due to sudden cardiac death and more frequently due to non-cardiac causes, mostly cancer [56, 57]. This shift to non-cardiovascular death can be observed especially in the elderly [21]. Rutten et al. analyzed the last year of 399 HF patients, with a mean age of 82.3 years at death. The mode of death was sudden death in 28%, progressive HF in 23%, and other causes in 49% [22]. Analyzing the cause of death by age after CRT implantation, a higher rate of non-cardiac causes was found among older patients, while the incidence of cardiac death was comparable between the age groups [18, 19, 29]. These findings suggest that non-cardiac death, due to the higher rate of multimorbidity, is the main factor in the survival difference between elderly and nonelderly patients.

Similarly, in our present analysis, there was a statistically significant increase in all-cause mortality in the older subgroups, which is an expected outcome in patients with an increased prevalence of coexisting chronic illnesses. These findings emphasize the importance of comorbidities, especially in CRT responder patients, since their long-term outcome will be mainly determined by non-cardiac death due to the presence of these concomitant conditions.

### Choice of device

The current guidelines recommend implantation of a CRT-D device primarily at a younger age as the cumulative rate of appropriate shocks (particularly in non-ischemic patients) is lower in older patients than in younger ones, which was confirmed by observational trials and other RCTs (e.g., the DANISH study) [30, 58, 59]. Moreover, no age-related difference was found in the risk of mortality after appropriate shock therapy [18, 30]. A post hoc analysis of the DANISH study revealed that in non-ischemic patients the association between ICD therapy and all-cause mortality decreased with advancing age in a linear relation and found no association between ICD and survival in patients over 70 years of age [60]. Although ischemic etiology is more common in older patients, the rate of malignant ventricular arrhythmias in the elderly and the relative risk reduction in the presence of comorbidities might suggest implantation of CRT-P alone as an alternative to CRT-D since, in older patients, prevention of sudden cardiac death has only a limited effect on all-cause mortality, which is mostly dominated by non-cardiac causes [58, 60–62]. Therefore, choosing the optimal device for the oldest patient population is a relevant issue. Although CRT-D implantation does not seem to mean an increased risk to the elderly, several studies have reported no benefit regarding morbidity and mortality compared to CRT-P alone [63–67]. In our patient population, we observed that the mean age of both CRT-P and CRT-D patients increased significantly since 2000, due to the development of new drugs for HF, longer life expectancy, and thus the higher number of older HF patients [68–70]. However, the mean age of CRT-P recipients was significantly higher than that of CRT-D recipients. This difference can be explained by guideline recommendations and the preferences of treating physicians and elderly patients. Even though the number of CRT-D implantations increased based on previous and our current study, implantation of CRT-D is still significantly lower in older patients in everyday clinical practice [18–20, 29, 31–33, 35, 36, 42].

## Conclusions

In this large-scale, real-world, retrospective study of patients who underwent CRT implantation, our results demonstrate that CRT is as effective in improving left ventricular ejection fraction and as safe in the elderly as in younger ones. Time-trend analyses showed an increase in the mean age of CRT-P and CRT-D patients with a significant difference between the two groups and in the percentage of CRT-D implantations. Moreover, response rate increased in each subgroup as a result of adding new effective pharmacological treatments.

## Limitations

The present study has several limitations. First, this study was a retrospective analysis from our single-center CRT database; consequently, echocardiographic evaluations were not performed by the same physician, which might have influenced the assessment of reverse remodeling. Second, we do not have data about the mode of death; thus we cannot investigate cardiac and non-cardiac causes separately. Third, not all of our patients have been followed at our center; thus data regarding procedure-related complications might be missing from our database, which might have influenced our results.

### Supplementary Information

Below is the link to the electronic supplementary material.

Supplementary file1 (DOCX 13 KB)

## Data Availability

The data that support the findings of this study are available from the corresponding author, [BM], upon reasonable request.
