# Supplementary material for: Long-term outcome of cardiac resynchronization therapy patients in the elderly
Source: GeroScience. 2023 Feb 17;45(4):2289–301. doi: 10.1007/s11357-023-00739-z (PMC10651580; doi:10.1007/s11357-023-00739-z)
Supplement: Supplementary file 1 — Supplementary file1 (DOCX 13 KB) [file 11357_2023_739_MOESM1_ESM.docx]

Supplementary tables are intended for publication as an online data supplement.

Supplementary Table 1. The mean age of CRT recipients by device type within 5-year intervals

|  | 2000-2004 | | 2005-2009 | | 2010-2014 | | 2015-2019 | |
| --- | --- | --- | --- | --- | --- | --- | --- | --- |
|  | CRT-D | CRT-P | CRT-D | CRT-P | CRT-D | CRT-P | CRT-D | CRT-P |
| Mean age | 60.6±9.9 | 62.7±11.7 | 63.2±10.4 | 65.8±10.1 | 65.7±10.4 | 69.9±9.5 | 67.8±9.4 | 73.2±9.6 |
| p-value | 0.22 | | <0.01** | | <0.01**** | | <0.01**** | |

*** p<0.01, **** p<0.0001*

Supplementary Table 2. The mean age of CRT-D and CRT-P recipients within 5-year intervals

|  | 2000-2004 | 2005-2009 | 2010-2014 | 2015-2020 | p-value |
| --- | --- | --- | --- | --- | --- |
| CRT-D mean age (yrs, ±SD) | 60.6±9.9 | 63.2±10.4 | 65.7±10.4 | 67.8±9.4 | <0.01**** |
| CRT-P mean age (yrs, ±SD) | 62.7±11.7 | 65.8±10.1 | 69.9±9.5 | 73.2±9.6 | <0.01**** |

***** p<0.0001*
